# Enhancing Force Control of Prosthetic Controller for Hand Prosthesis by Mimicking Biological Properties

**DOI:** 10.1109/JTEHM.2023.3320715

**Published:** 2023-09-29

**Authors:** Qi Luo, Minglei Bai, Shuhan Chen, Kai Gao, Lairong Yin, Ronghua Du

**Affiliations:** School of Automotive and Mechanical EngineeringChangsha University of Science and Technology 12418 Changsha 410114 China; School of Biomedical SciencesThe Chinese University of Hong Kong 26451 Hong Kong 999077 China

**Keywords:** Electromyography (EMG), prosthetic control, biomimetic model, neuromorphic computation, force control

## Abstract

Prosthetic hands are frequently rejected due to frustrations in daily uses. By adopting principles of human neuromuscular control, it could potentially achieve human-like compliance in hand functions, thereby improving functionality in prosthetic hand. Previous studies have confirmed the feasibility of real-time emulation of neuromuscular reflex for prosthetic control. This study further to explore the effect of feedforward electromyograph (EMG) decoding and proprioception on the biomimetic controller. The biomimetic controller included a feedforward Bayesian model for decoding alpha motor commands from stump EMG, a muscle model, and a closed-loop component with a model of muscle spindle modified with spiking afferents. Real-time control was enabled by neuromorphic hardware to accelerate evaluation of biologically inspired models. This allows us to investigate which aspects in the controller could benefit from biological properties for improvements on force control performance. 3 non-disabled and 3 amputee subjects were recruited to conduct a “press-without-break” task, subjects were required to press a transducer till the pressure stabilized in an expected range without breaking the virtual object. We tested whether introducing more complex but biomimetic models could enhance the task performance. Data showed that when replacing proportional feedback with the neuromorphic spindle, success rates of amputees increased by 12.2% and failures due to breakage decreased by 26.3%. More prominently, success rates increased by 55.5% and failures decreased by 79.3% when replacing a linear model of EMG with the Bayesian model in the feedforward EMG processing. Results suggest that mimicking biological properties in feedback and feedforward control may improve the manipulation of objects by amputees using prosthetic hands. Clinical and Translational Impact Statement: This control approach may eventually assist amputees to perform fine force control when using prosthetic hands, thereby improving the motor performance of amputees. It highlights the promising potential of the biomimetic controller integrating biological properties implemented on neuromorphic models as a viable approach for clinical application in prosthetic hands.

## I. Introduction

The dilemma of individuals with amputations encountering hand function impairment has spurred innovations in prosthetic technologies. However, while significant progress in the development of prosthetic hands in recent years, a formidable challenge remains in the form of consistently high rejection rates among users [1], [2]. A critical and persistent issue with conventional prosthetic hands lies in their inability to exhibit compliant properties in interacting with objects. This deficiency means that these prosthetic hands struggle to adapt effectively to the varying stiffness or fragility of objects they come into contacting with, limiting their utility and naturalness in daily activities. An example is that an amputee would command a prosthetic hand to grasp — in almost the same manner — a boiled egg and a raw egg, because it is difficult for the amputee to discern the differences in mechanical subtlety [3], [4], [5], [6]. Consequently, a critical consideration in advancing beyond conventional prosthetic hands involves the practical implementation of principles that enable the device to replicate an anthropopathic compliant control.

In the realm of prosthetic devices designed for interacting with real-world objects, particularly those with deformable and delicate characteristics, a fundamental requirement is for these devices to seamlessly adapt their actions to suit the specific attributes of the objects they manipulate. In individuals with intact limbs, the nuanced understanding of object properties arises from the intricate interplay of visual, tactile, and proprioceptive sensory inputs [7]. However, the situation is markedly different for individuals with amputations who contend with compromised tactile and proprioceptive feedback at the sites of limb loss, necessitating their primary reliance on visual cues to indirectly assess the exerting forces. An emerging avenue for elevating the functionality of prosthetic hands involves the engineering of proprio-sensor signals into biomimetic formats, such as spike trains. This innovative approach holds the promise of replicating the natural flow of proprioceptive information at a spinal-level control, thus facilitating the implementation of biomimetic control. Importantly, this paradigm shift stands in stark contrast to the conventional control that typically govern the field of robotics.

Computational models are central to the integration of neural principles in human sensorimotor control for prosthetic control. Even though our understanding of voluntary and reflexive control of movement is limited due to difficulties of neural recording in human, mammalian experiments reveal what thought to be instrumental for sensorimotor control: motor units with patterned recruitment order [8], spinal-level neural circuitry [9], dynamics of muscle spindle [10], muscle with viscoelastic properties [11] and the list goes on. Take proprioception as an example, a systematic unfolding for its understanding and application includes the following endeavors: pioneering work for spindle neurography [10], its computational modeling and continuing refinement [12], [13], [14], the engineering improvement for its real-time emulation and spiking behavior [15], and recently a “model-in-the-loop” application that demonstrated its feasibility for limb control [16]. On the ground of human sensorimotor control and the computational models thereof, we hypothesize that using biomimetic models for prosthetic control would enhance the overall performance.

Another substantial hurdle in prosthetic control revolves around the precise interpretation of intended movements of individuals with amputations. To address this challenge, electromyography (EMG) signals, directly sourced from the motor cortex, have emerged as a versatile tool in a wide array of control strategies for prosthetic hands. These strategies encompass proportional control [17], [18], [19], [20], [21], regression control [22], [23], [24], on-off control [25], finite state machine control [26], [27], pattern recognition-based control [28], [29], [30], [31], [32], postural control [33], [34], [35], etc. Techniques of motor unit decomposition [36], [37] provided the possibility to drive artificial muscles with an abundance of motor unit activity. However, the decomposition algorithm is yet fast enough for real-time applications, and existing actuators do not have the resolution of individual muscle fibers, which inevitably obscures the motor unit action potential (MUAP) decoded from EMG.

An innovative approach to myocontrol involves the exploration of biological-inspired models for decoding latent control variables within raw EMG signals. In general, EMG signals are conceptualized as amplitude-modulated band-limited noise [38], [39], necessitating the use of specific algorithms to filter these signals and extract their amplitude envelopes for myocontrol [40]. A novel Bayesian model for EMG has been introduced, portraying motor intent as a stochastic process characterized by abrupt transitions [41]. This approach reflects a growing trend in harnessing nature-inspired insights to enhance the precision and sophistication of myoelectric control. The Bayesian model does not contain the motor-unit level detail of human sensorimotor system, but it operates on the biological principle that force generation can be discontinuous due to sudden recruitment of new motor units. Subsequent work showed advantages in myocontrol applications using Bayesian model of EMG [42].

Our prior study has suggested the potential of mode-based controller for mimicking human hand control [16], which may lead to a unique approach for prosthetic control among many other strategies. Moreover, the force-control capability and functional grasping performance of the biomimetic control was evaluated, which demonstrated the applicability and potential of biomimicry for prosthetic hand control [43], [44]. These studies established that the biomimetic controller possesses human-like capabilities for force and stiffness control, which are fundamental qualities of human sensorimotor system.

In order to further investigate the effect of feedforward EMG decoding and proprioception on the biomimetic controller, and explore its utility for guiding future design of prosthetic hands, we examined two factors in the model-based closed-loop controller: 1) model for proprioceptive feedback, and 2) model for feedforward EMG decoding. For each factor, was tested how single-finger performance could be affected by replacing a simple linear model with a biomimetic counterpart. If proven that even a partially biomimetic implementation either in the feedforward or the feedback pathway, despite their computational complexity, could translate stump EMG to accomplish tasks with quantifiable improvements, then it will provide an important foundation toward a full-fledged, biomimetic, prosthetic hand.

## II. Materials and Methods

### A. Construction of Tendon-Driven Prosthetic Hand

In order to evaluate the possible benefit of adopting biomimetic principles in feed-forward and feedback pathway of the biomimetic controller, we implemented a testbed that included a model-based controller with a tendon-driven prosthetic hand.

**FIGURE 1.**
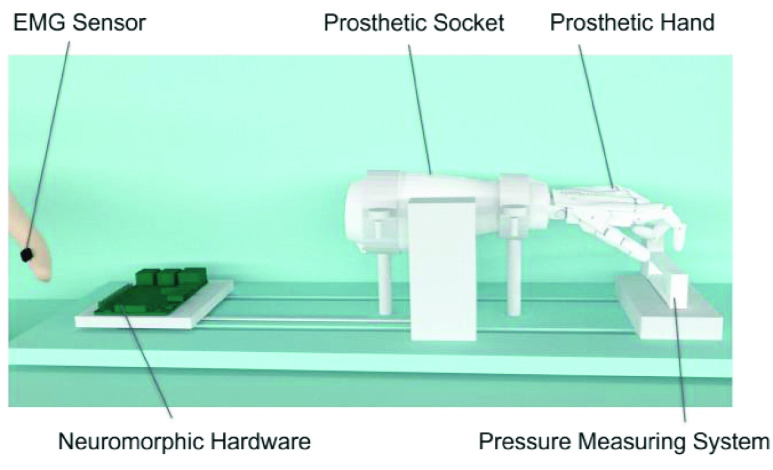
The design of a tendon-driven prosthetic hand actuated by a torque motor, which executes commands issued from the stump EMG modulated by neuromorphic hardware. The torque motor is integrated into the prosthetic socket. A force transducer measures the downward pressure from the index finger.

The mechanical part of the prosthetic hand was 3D-printed using the InMoov open-source design [45]. Joints in the hand were pulled using polyethylene (PE) cable as proxies of human tendon, with each tendon simultaneously flexing the metacarpophalangeal (MCP), distal interphalangeal (DIP), and proximal interphalangeal (PIP) joints. Each tendon was attached to the shaft of a torque motor (PD2-C42, Nanotec Electronics GmbH & Co.KG, Germany) communicated through a CANopen interfacing card (ECAN-IT, Guangcheng Technology Co., Ltd., Shenyang, China), and the motor generated a rotational torque resulting in a tension on the tendon. In this study, only flexion about the finger was tendon-actuated; finger extension was achieved by installing springs in the joints to maintain a torque for hand opening. The illustration of the tendon-driven prosthetic hand system is depicted in Figure 1.

In this study, we only connected the tendon for flexion to the index finger of the prosthetic hand, leaving all other fingers unactuated. The EMG signals from flexor carpi ulnaris provided the source of control for finger flexion. If all five tendons are connected to the torque motor, activating the EMG will close the hand and therefore create a grasping movement. A universal prosthetic socket was constructed for all participants, and it could be customized for amputees by utilizing a forearm adapter to accommodate variations in arm length.

### B. Model-Based Controller

Here we concentrate on the spinal level of human nervous system because the supra-spinal structures are not lesioned through amputation. For human hand control, a representative joint like the metacarpophalangeal joint operates through the coordinated action of antagonistic muscles. The muscle contraction generates precise and controlled forces, which are meticulously transmitted through tendons, ultimately serving to finely manipulate the motion of joint. The level of muscle contraction is determined by the spiking excitations the muscles received from alpha and gamma motoneurons, as well as the proprioceptive feedback provided by muscle spindles. Therefore, the monosynaptic spinal loop forms a closed-loop for regulating muscle tone and reflex.

**FIGURE 2.**
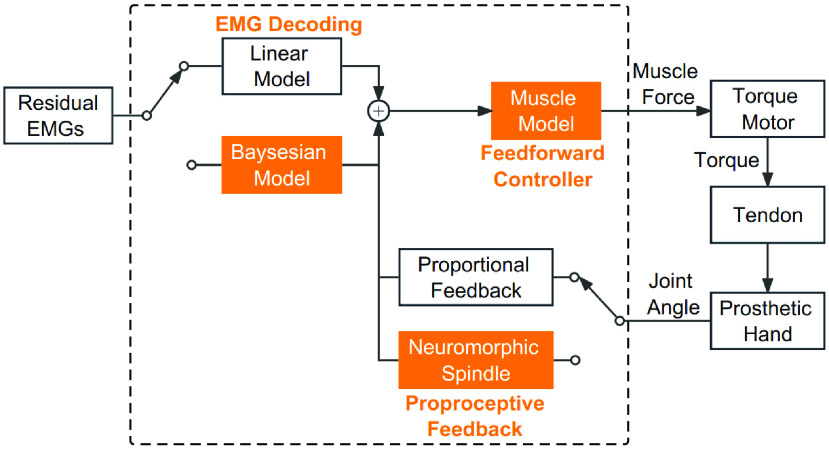
Control flow diagram of the model-based closed-loop controller for the tendon-driven prosthetic hand. Two factors were tested in the experiment: 1) Proprioceptive feedback, with two options of proportional feedback and neuromorphic spindle; 2) Feedforward EMG decoding, with two options of linear model and Bayesian model.

Figure 2 illustrates the architecture of controller for the prosthetic hand. The amputee activates the wrist flexor, from which the EMG was captured and decoded into the alpha motor command to a feedforward controller. This architecture allows for the feedforward controller to be implemented a variety of muscle models as resource permits. The muscle model implemented in this system is the Hill-type muscle model [46]. The muscle model converts alpha-motoneuron spikes into muscle force, depending on the muscle’s temporal length and lengthening velocity. Active force is caused by the contractile elements in a muscle through the actin and myosin ratcheting mechanism. The active force has been scaled with respect to the length of muscle in the muscle model. Feedback on muscle length was calculated from the shaft rotation of torque motor, and subsequently the length was transformed through a model of proprioceptor. The spindle model senses changes in the muscle length, and then adjusts the output force of the neuromorphic chip in real time. Afferents were adapted with spiking interfaces as described in [15], which is a key feature of neuromorphic computation. Closed-loop control was achieved by superimposing the EMG command and the proprioceptive feedback. The main control loop was coordinated on a PC (Intel Core i7-8700CPU, 3.20 GHz, 16 GB Memory, Microsoft Windows 10 64-bit) at 100 Hz sampling rate.

We used programmable very-large-scale-circuit (VLSI) hardware to implement simple models of spiking neurons, muscle model, and muscle spindle proprioceptor. For the neuromorphic chip, all spiking neuron behavior was generated autonomously and in real-time by a low-cost FPGA (Xilinx Spartan-6). It emulated 6 motoneuron pools with 768 spiking neurons, 6 Hill-type muscle fibers, and 1 muscle spindle projecting 128 spiking. This setup had multiple parallel proprioceptive closed-loop pathways, resembling the concurrent monosynaptic pathways in human spinal cord.

### C. Subjects

Three non-disabled subjects and three forearm amputee subjects (all male, age range: 50–54 yrs) participated in the study. The detailed descriptions of amputee subjects are contained in Table 1. All six participants are right-handed. Subjects had no history of neurological disorders. This study was approved by the Ethics Committee of Human and Animal Experiments of the Med-X Research Institute of Shanghai Jiao Tong University. Prior to participating in the study, all participants provided written consent.

**TABLE 1:** Clinical Information of Amputees

Subject	Age	Sex	Amputation level and side	Years since amputation	Myoelectric prosthesis experience
S1	53	M	Left Distal third of left forearm (long residual limb)	2	Yes
S2	50	M	Left Distal third of left forearm (long residual limb)	34	No
S3	54	M	Right Distal third of right forearm (long residual limb)	7	Yes

### D. Experimental Setup and Force-Control Task

**FIGURE 3.**
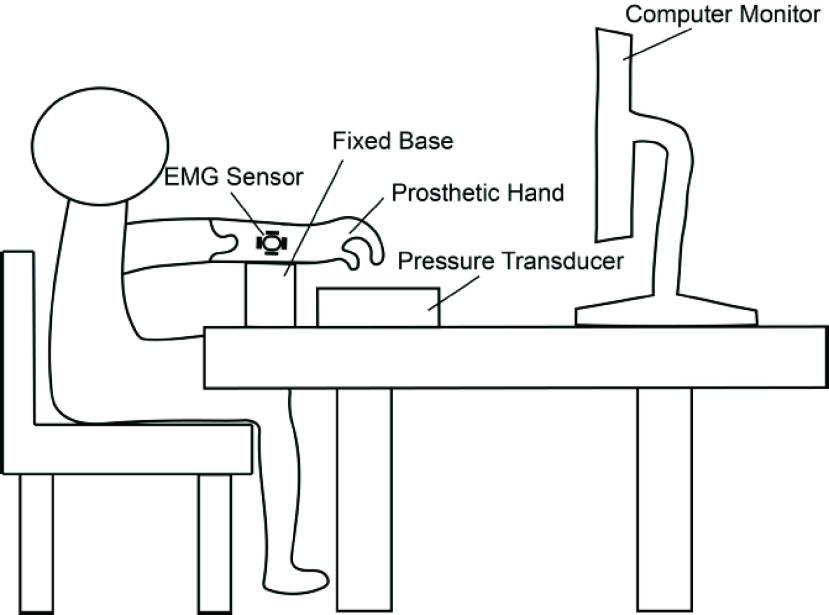
The experimental setup for the single-finger force control test. A host computer was in the loop to acquire EMG, decode the alpha motor command from EMG, interact with neuromorphic spindles, and issue the calculated commands to the torque motor. Subjects were free to look at either the screen or the finger during experiment.

In the experiment (Figure 3), subjects were seated comfortably in a chair, with a computer monitor positioned at a distance of approximately 60 cm from them. The surface EMG signals from the flexor carpi ulnaris (FCU) were recorded using the Delsys system (Trigno $^{\mathrm{ TM}}$ Wireless EMG System, Delsys Inc., US) at a sampling rate of 2000 Hz. Concurrently, the index finger of the prosthetic hand exerted pressure on a force transducer (Model FNA, 0~30 N, Forsentek Co., Ltd., Shenzhen, China), which recorded the force data at a rate of 100 Hz with a 12-bit resolution (Model USB-201, Measurement Computing Corp., MA, US).

**FIGURE 4.**
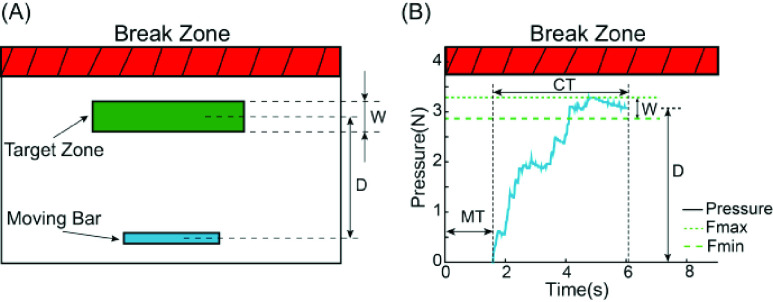
(A) The visual interface for the “press-without-break” task. The height of the moving bar directly mirrored the applied finger pressure. The primary aim of the task was to elevate the moving bar and maintain it within the target zone for a continuous duration of 1 second. Task complexity increased as the distance (D) grew longer and the width (W) became narrower. A vivid red break zone was clearly marked, symbolizing potential object breakage upon entry. Participants were explicitly directed to steer clear of this zone at all times. (B) An illustrative successful trial. Starting from the initiation of movement (t = 0), the participant spent MT seconds to traverse a 1cm distance until the fingertip made contact with the transducer. Following this contact at t = MT, the participant required CT seconds to maintain the force within the ( $\text{F}_{\mathrm {min}}$, $\text{F}_{\mathrm {max}}$) range for a continuous 1-second duration.

Participants were given specific instructions to perform a sequence of “press-without-break” tasks. A monitor displayed a moving bar, with its height representing the finger pressure as detected by the transducer. In each trial, participants were tasked with swiftly raising the moving bar to a designated green target zone. This objective was achieved by activating the wrist flexor muscles to flex the index finger of the prosthetic hand (Figure 4A). At the commencement of each trial, the finger began at a default position, situated 1 cm above the force transducer. Therefore, the finger had to traverse this 1 cm distance, make contacting with the transducer, and then carefully manage the pressure to fulfill the task requirements.

A trial was deemed successful when the moving bar remained within the target zone for a continuous duration of 1 second. Positioned above the target zone was a prominently red break zone, as depicted in Figure 4A. Participants were explicitly informed that encroaching into this break zone would result in the virtual object being shattered, and they were strongly cautioned against such actions. In this task, we established a defined breakage threshold, marking the lower boundary of the break zone, at 4.4 N. The designated breakage threshold exceeded the upper bound of the highest target zone by roughly 30% [43]. A trial was considered unsuccessful if it reached a duration of 15 seconds or if object breakage was detected. The force-time profile of a successful trial is illustrated in Figure 4B. Essentially, the “press-without-break” task was introduced to gauge the ability to promptly and precisely generate the expected force while adhering to the constraints imposed by the brittleness of virtual object. Participants received continuous visual feedback throughout the task to assist them in achieving these objectives.

For each trial, the index of difficulty (ID) is calculated as $ID=\log _{2}(2D/W)$ [43], [47].The execution of the task is characterized by the correlation between ID and completion time (CT), representing the time taken to accomplish the task. The experiment encompassed six distinct IDs, and Table 2 provides the corresponding target distances (D) and target widths (W) for each ID. We focus on the following metrics for quantification of performance in the “press-without-break” task.

1. Success rate (SR): the ratio of count between successful trials and total trials.
2. Break rate (BR): the ratio of count between failed trials (due to object breakage) and total trials.
3. Throughput (TP): an outcome metric for assessing the speed-accuracy relationship, defined as [43]:
\begin{equation*} TP=\frac {1}{N}\sum \limits _{i=1}^{N} {ID_{i}} /CT_{i}\end{equation*}
4. Index of performance (IP): the inverse of slope between CT and ID [48].

**TABLE 2:** Catalog of Distances (D) and Widths (W) Matched With Respective Indices of Difficulty (ID)

D (N)	W (N)	ID (bits)
1.8	0.1	5.17
1.8	0.2	4.17
1.8	0.3	3.59
3.1	0.1	5.95
3.1	0.2	4.95
3.1	0.3	4.37

### E. Factors Tested With the Model-Based Controller

Two factors were tested in the force-control experiment, one being the model for EMG decoding, and the other the model for proprioceptive feedback. The main hypothesis is that switching to biomimetic models in the controller would enhance the performance of prosthetic hand in the force-control task.

*Factor 1: Feedforward EMG decoding*

**FIGURE 5.**
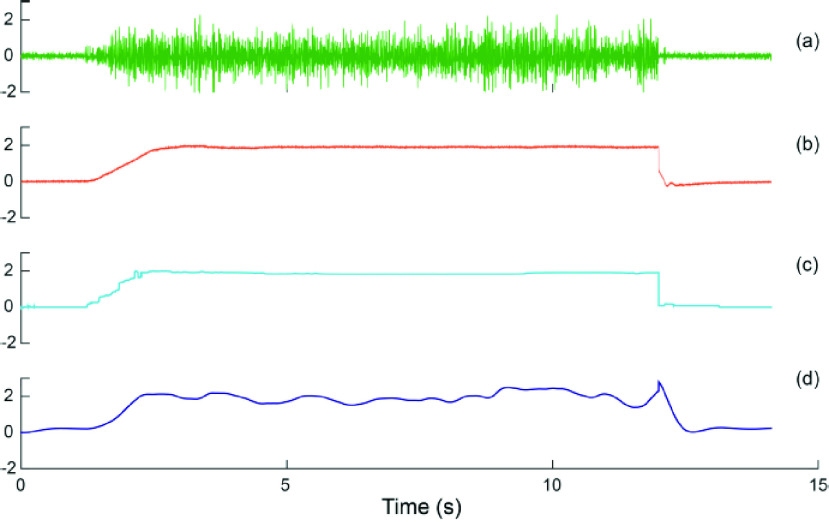
Comparison between EMG models when filtering of a sample snippet of raw EMG. (a) raw EMG signal (in mV) of biceps brachii; (b) torque (in percentage maximum voluntary contraction (MVC) with flexion upward); (c) nonlinear Bayesian filter applied to the rectified EMG signal; (d) linear Butterworth filter applied to the rectified EMG signal with cutoff at 1 Hz.

The experiment first tested the effect of EMG decoding in the feedforward branch of the controller. Two models were compared: a plain linear filter (3rd order Butterworth low-pass filter, cut-off frequency 1 Hz) and a Bayesian nonlinear filter as formulated in Sanger ([41], $\alpha $ = 1e – 4, *ß* = 1e −18, 128-level quantization) (Figure 2). The performance between the two filters is visualized in Figure 5; refer to [41] for detailed analyses. Both feedforward models were applied on rectified EMG signals from the flexor carpi ulnaris of the amputee. The Bayesian nonlinear filter chose Exponential Distribution from 3 prior distributions since it best describes the composition of EMG amplitude; in addition, the Bayesian nonlinear filter incorporated a Poisson jumping process that explicitly characterized the intention of quickly moving to a target. Under both circumstances, the Bayesian nonlinear filter adopted more biomimetic features than the plain linear filter. Figure 5 shows a qualitative comparison of EMG models when filtering a snippet of biceps EMG, a better model should produce an outcome signal visually resembling the torque.

*Factor 2: Proprioceptive Feedback*

The second factor tested in the experiment was the proprioceptive feedback. Also two models were tested: a linear proportional feedback that converted muscle length to motor command, and a biomimetic spindle that obeys the nonlinear properties in proprio-sensing. In both models, the feedback gain was set such that 10% of muscle stretch elicited approximately 1% change in the motor command. For the spindle model, in particular, the gamma dynamic and gamma static inputs were both set to 40 pps, which did not include complex interactions such as alpha-gamma co-activation, but it still kept the spindle active on a moderate level.

The combination of the two factors yielded four possible conditions:

1) Linear EMG decoding, proportional feedback;
2) Linear EMG decoding, neuromorphic spindle;
3) Bayesian EMG decoding, proportional feedback;
4) Bayesian EMG decoding, neuromorphic spindle.

All conditions were tested in a randomized block design.

### F. Experimental Protocols

Each subject participated in a single-visit experiment containing four blocks, each of which covered a combination of the two factors described above. In each block, the subject received a total of 36 trials with 6 IDs and 6 repetitions of each. Therefore, a subject was requested to accomplish 144 trials in the entire experiment. There was a five-minute break between adjacent blocks, and adhoc breaks were allowed at any time the subject wanted to rest. Each visit took approximately an hour and a half. Both the sequence of blocks and the sequence of within-block trials were randomized. Visualization and data collection were developed using Unity 3D (version 2019, Unity Technologies, CA).

### G. Statistical Analysis

Two-way repeated measures ANOVA was conducted using R (version 4.1.0) to evaluate the effects of two factors on the force-control task, specifically on success rate, break rate, throughput as defined above. Other data processing was done using Matlab (R2020b, MathWorks Inc., Natick, MA). The significance level in all statistical and correlation analyses was set at ${p} < 0.05$.

## III. Results

We analyzed whether adopting biomimetic models in the controller produced any benefit for the force control in a single prosthetic finger. Differences in performance between non-disabled subjects and amputees potentially reflect changes in the ability of sensorimotor control after amputation.

### A. Effects of Biomimetic Control in Non-Disabled Subjects

**FIGURE 6.**
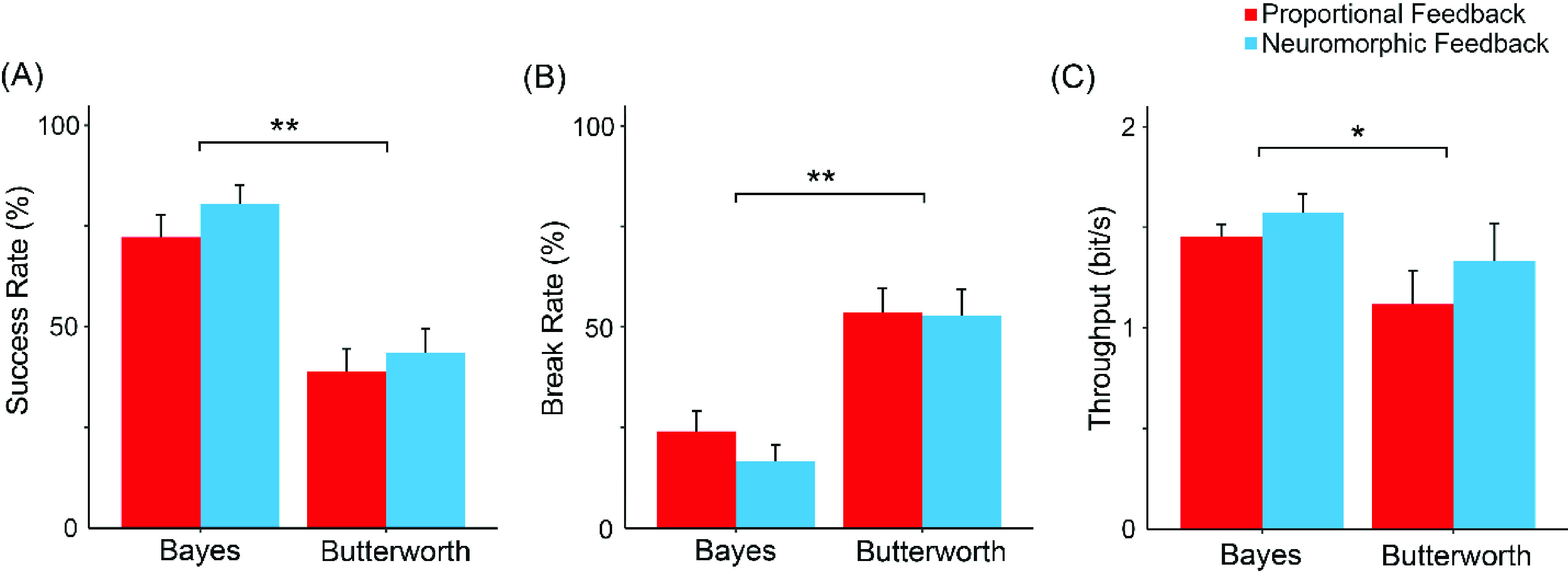
Performance metrics of non-disabled subjects. (A) Success Rate, (B) Break Rate, (C) Throughput. ( $^{\ast} $, $p < 0.01$; $^{\ast \ast }$, $p < 0.001$).

In the 3 non-disabled subjects (Figure 6), data showed that the mean of success rate (SR) increased by 11.5% with a neuromorphic spindle (62.0%±29.2%), compared with linear proprioceptive feedback (55.6% ± 28.7%), but the main effect of proprioception was not significant. Furthermore, SR significantly improved by 85.4% when the EMG was decoded using Bayesian nonlinear model (76.4% ± 21.6%) compared with a linear model (Figure 6A, 41.2% ± 24.4%, main effect of feedforward EMG decoding, F(1,68) = 41.703, $p < 0.001$). Interaction was non-significant (F(1,68) = 0.116).

The occurrence of object breakage (break rate, BR) was 9.5% lower with a neuromorphic spindle (Figure 6B, 35.2% ± 29.2%, F(1,68) = 0.463, $p$ = 0.499), compared with proportional feedback (38.9% ± 27.6%), but the main effect of proprioception was not significant. Moreover, BR significantly decreased by 62% with Bayesian nonlinear model for EMG decoding (20.4% ± 19.6%), compared with a linear model (53.7% ± 25.9%, main effect of feedforward EMG decoding F(1,68) = 37.469, $p < 0.001$).

The statistical pattern with throughput (TP) is similar with that of SR. Replacing the linear model (1.227 ± 0.740 bits/s) with Bayesian nonlinear model significantly improved TP by 23.3% (Figure 6C, 1.513 ± 0.338 bits/s, main effect of feedforward EMG decoding F(1,68) = 4.445, $p < 0.05$). No significance was found for the main effect of proprioception on TP in non-disabled subjects.

**FIGURE 7.**
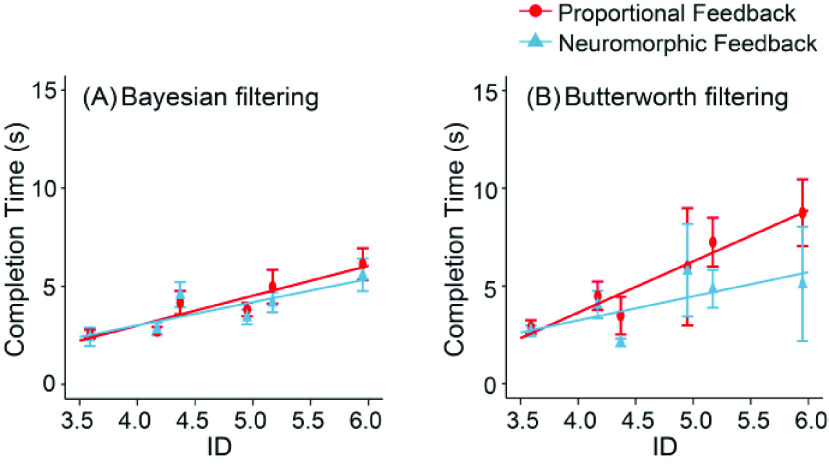
The linear relationship between completion time (CT) and index of difficulty (ID) for non-disabled subjects. (A) Bayesian filtering (proportional feedback: ${y}$ = $1.53{x}$- 3.11, $R^{2}$ = 0.874, ${p} < 0.05$; neuromorphic spindle: ${y}$ = $1.19{x}$- 1.74, $R^{2}$ = 0.705, ${p} < 0.05$), and (B) Butterworth filtering (proportional feedback: ${y}$ = $2.4{x}$- 6.33, $R^{2}=0.665$, ${p} < 0.05$; neuromorphic spindle: ${y}$ = $1.23{x}$- 1.69, $R^{2}$ = 0.506, ${p}$ = 0.113). The solid red lines indicate the proportional feedback and the solid green line indicate the neuromorphic feedback.

The linear relationship between CT and ID for non-disabled subjects is shown in Figure 7. As can be seen, when using Bayesian model for EMG decoding (Figure 7A), the relationship is almost not affected by model of proprioception, as is the case suggested by index of performance (IP_prop_ = 0.654 bits/s; IP_neuro_ = 0.840 bits/s). When using a linear model for EMG decoding (Figure 7B), non-disabled subjects show decreased slope in the CT-ID relationship and increased index of performance (IP $_{\mathrm {prop}}=0.417$ bits/s; IP_neuro_ = 0.813 bits/s) when the feedback switched to a neuromorphic spindle.

### B. Effects of Biomimetic Control in Amputees

**FIGURE 8.**
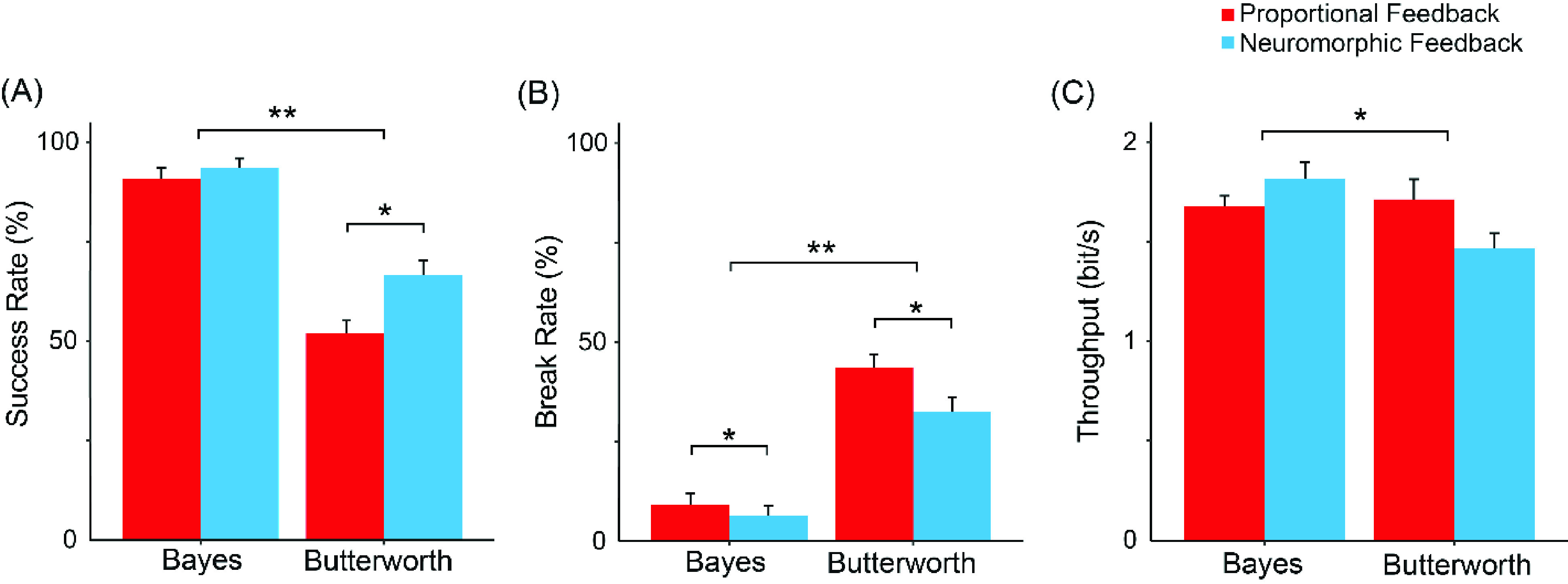
Performance metrics of amputee subjects. (A) Success Rate, (B) Break Rate, (C) Throughput. ( $^{\ast} $, $p < 0.01$; $^{\ast \ast }$, $p < 0.001$).

In 3 amputees (Figure 8), two-way repeated measures ANOVA showed that the SR was significantly improved by 12.3% when proprioceptive feedback was provided using the biomimetic spindle (80.1%±18.6%), compared with a simple linear model (Figure 8A, 71.3% ± 23.1%, main effect of proprioceptive feedback, F(1,68) = 8.857, $p < 0.01$). More prominently, SR was significantly improved by 55.3% when the EMG was decoded using Bayesian nonlinear model (92.1% ±10.9%) compared with a linear model (59.3% ± 26.1%, main effect of feedforward EMG decoding, F(1,68) = 123.617, $p < 0.001$). The interaction was significant (F(1,68) = 4.147, $p < 0.05$). Tukey post-hoc test showed that SR was significantly increased with a biomimetic spindle, only when EMGs were decoded using linear model, but not using Bayesian model.

Mean of BR was significantly lower by 26.5% when proprioceptive feedback was provided using the neuromorphic spindle (19.4% ± 18.5%), compared with a proportional feedback model (Figure 8B, 26.4% ± 21.6%, main effect of proprioceptive feedback, F(1,68) = 5.066, $p < 0.05$). BR was also significantly decreased by 79.2% with Bayesian nonlinear model for EMG decoding 7.9% ± 10.9%), compared with a linear model (38.0% ± 15.7%, main effect of feedforward EMG decoding, F(1,68) = 95.132, ${p} < 0.001$).

TP achieved a remarkable 10.2% enhancement when utilizing Bayesian nonlinear model for EMG decoding (1.745 ± 0.297 bits/s) compared to the linear model (Figure 8C, 1.584 ± 0.388 bits/s, main effect of feedforward EMG decoding F(1,68) = 4.122, $p < 0.05$). However, it did not significantly increase TP by replacing the proportional feedback (1.689 ± 0.335 bits/s) with a neuromorphic spindle (1.640 ± 0.373 bits/s). The interaction term was significant (F(1,68) = 5.618, $p < 0.05$), which warranted a Tukey post-hoc test showing that TP was significantly increased when switching to Bayesian model for EMG decoding, only under feedback provided by neuromorphic spindle, but not proportional feedback.

**FIGURE 9.**
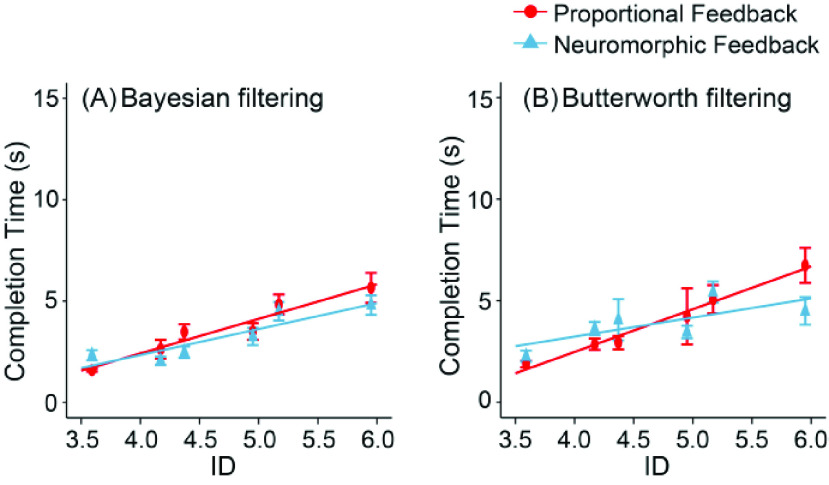
The linear relationship between completion time (CT) and index of difficulty (ID) for amputee subjects. (A) Bayesian filtering (proportional feedback: $y$ = $1.62x$ – 4.05, $R^{2}=0.815$, ${p} < 0.001$; neuromorphic spindle: $y$ = $1.13x$ – 2.23, $R^{2}=0.672$, ${p} < 0.01$) and (B) Butterworth filtering (proportional feedback: $y$ = $2.04x$ – 5.51, $R^{2}=0.913$, ${p} < 0.001$; neuromorphic spindle: $y$ = $1.07x$ – 1, $R^{2}=0.483$, ${p} < 0.05$).

The linear relationship between CT and ID for amputees is shown in Figure 9. As can be seen, when using Bayesian model for EMG decoding (Figure 9A), the speed-accuracy relationship is improved by a neuromorphic spindle for proprioception, represented by higher index of performance (IP_prop_ = 0.617 bits/s; IP_neuro_ = 0.885 bits/s). When using a linear model for EMG decoding (Figure 9B), the trends are similar with increased index of performance (IP_prop_ = 0.490 bits/s; IP $_{\mathrm {neuro}}=0.935$ bits/s) when the feedback loop switched to a neuromorphic spindle.

## IV. Discussion

In the study, we evaluated the effect of feedforward EMG decoding and proprioception to the force control capability of the biomimetic controller in a tendon-driven prosthetic hand. A “press-without-break” task was designed to assess the precision and sensitivity of force control. All amputees and non-disabled subjects were able to complete the task. Compared in all categories of performance indices, the prosthetic controller with biomimetic models consistently outperformed the one with linear models in the force-control experiment. Our results supported the hypothesis that mimicking biological properties either in the feedforward or feedback control may enhance the manipulation of objects by amputees using prosthetic hands.

The study presented a detailed quantification of the ability of the prosthetic hand in force generation and regulation, which provided a way to explore the potential of biomimicry for prosthetic control. In particular, since EMG decoding and proprioceptive information are crucial in brain-hand-environment interaction [49], we hypothesize that a prosthetic hand equipped with neuromuscular-like properties would better execute human intention in manipulation tasks. This hypothesis is supported by our data that the performance of amputees in a “press-without-break” task indeed improved with biomimetic models. Both non-disabled subjects and amputees showed similar trends, suggesting that the prosthetic control is compatible between the two populations. This study verified the benefit of adopting biomimetic computational models either in the feedforward EMG decoding or proprioception of the prosthetic controller. Our findings indicate that physiological principles of sensorimotor control conduce to the for prosthetic control, as even a partially biomimetic implementation can enhance force performance in prosthetic hand.

In the feedback loop of the controller, our data showed benefits of replacing simple proportional feedback with a neuromorphic muscle spindle. According to the theory of impedance control [50], having a human-like spindle improves the quality of the prosthetic hand as an impedance manipulator. In specific, the “press-without-break” task in fact creates an object with virtual stiffness, which accepts a pressure and responds in its deformation on visual display. When interacting with such objects (admittance), the theory of impedance control states that an amputee must be able to specify desired kinematics (flow) for the prosthetic hand, which turns into a force (effort) and eventually a desired impedance. Following this train of thought, the neuromorphic spindle makes it easier to specify desired lengthening of muscle using EMG; because of this, the prosthetic hand now enjoys greater ability to establish the desired impedance. Although the exact mechanism remains to be rigorously tested, our data support the explanation based on impedance control.

Another interesting finding was that the “press-without-break” task was almost isometric during force generation, and therefore the contribution of proprioception should be minimal as suggested by several human studies [51], [52]. In our case, however, the length of the virtual muscle was still allowed to change during the task (i.e. not strictly isometric) because of stiffness of the tendon, or deformation of the force transducer. It is likely that subtle changes in muscle length have been picked up and amplified by the neuromorphic spindle, which carries richer information than the proportional feedback. In general, our results have demonstrated that even though the changes in muscle length were small, human-like proprioception may still benefit the performance due to the rich information captured by spindle [53]. Moreover, physiological studies showed that human spindles are indeed recruited during isometric contractions [54], possibly due to alpha-gamma coactivation [55]. As a result, mechanisms that allow for spindle recruitment in quasi-isometric conditions should be included in future models.

When using the neuromorphic spindle for proprioception, the increase in success rate for amputees was more prominent only when the EMG was decoded with the plain linear model, but not the Bayesian model (Figure 8A, Tukey post-hoc results). One explanation is that the linear EMG model restricted the bandwidth of information exchange, therefore the bandwidth could be complemented by including a neuromorphic spindle in the feedback loop. However, when using Bayesian models of EMG, the bandwidth was already expanded in the feedforward branch, so the effect of neuromorphic spindle would not be as prominent.

Another explanation for the observed improvements in performance with biomimetic models is that they maintain a neuro-compatibility between the prosthetic device and amputees [56]. Alpha motor commands from the peripheral nervous system are supposed to activate a machinery with muscle properties, spindle feedback, etc. A controller with neuromorphic models, therefore, provided such a virtual environment that is compatible with the original intention of subjects before amputation. It is also noteworthy that all neuromorphic models implemented in this study are, in essence, mathematical formulations of high-order nonlinear systems [12], [14], [16]. Biomimetic models do not contradict the linear ones in principle of control, but rather they showed where the complexity should reside.

The main limitation of this study is that only flexion about the finger was tendon-actuated, while leaving the extension passively stretched by a spring. However, this approach lacks biological realism since both flexion and extension should ideally be separately actuated by a pair of antagonistic muscles. Furthermore, the flexion-only setup requires a constant EMG activity whenever the amputee wants to exert force, which means that the capability of resetting the resting posture is lost. To attain a more advanced level of biomimicry and effectively tackle the practical issue of posture resetting, it may be imperative to facilitate the operation of two autonomous muscles for each joint. By doing so, the controller can provide independent control of both flexion and extension, allowing for more realistic movements and the ability to reset the resting posture. In addition to enhancing the functionality of prosthetic devices, this modification also delivers a more natural and intuitive user experience. In the subsequent work, we will refine the prosthetic hand apparatus and biomimetic models to address the experimental limitations and further validate the potential advantages of model-based biomimetic control in clinical application.

## V. Conclusion

We evaluated the effect of feedforward EMG decoding and proprioception to the force-control capability of the biomimetic controller in a “press-without-break” task. Results showed that the prosthetic controller with biomimetic models outperformed the one with linear models. All outcome metrics weighted advantages of controller with the biomimetic models compared to the linear models. The study demonstrates that mimicking biological properties either in the feedforward or feedback control may enhance the manipulation of objects by amputees using prosthetic hands. It presents promising potential of our proposed biomimetic controller incorporating biological properties implemented on neuromorphic models as a practical avenue for clinical application to improve the performance of prosthetic hand control.
